# Clinical Utility of Bronchoalveolar Lavage Neutrophilia and Biomarkers for Evaluating Severity of Chronic Fibrosing Interstitial Lung Diseases

**DOI:** 10.7759/cureus.42162

**Published:** 2023-07-19

**Authors:** Benhur Joel Shadrach, Naveen Dutt, Poonam Elhence, Mithu Banerjee, Nishant Kumar Chauhan, Ram N Jalandra, Mahendra Kumar Garg, Pawan Garg, Abhishek Tandon, Saumya Shishir, Rishabh Kochar, Bhavesh Chhatwani, Piyush Pareek, Anika Parrikar

**Affiliations:** 1 Department of Pulmonary Medicine, All India Institute of Medical Sciences, Jodhpur, IND; 2 Department of Pathology, All India Institute of Medical Sciences, Jodhpur, IND; 3 Department of Biochemistry, All India Institute of Medical Sciences, Jodhpur, IND; 4 Department of Pulmonary Medicine, All India Institute of Medical Sciences, Bathinda, IND; 5 Department of Medicine, All India Institute of Medical Sciences, Jodhpur, IND; 6 Department of Radiology, All India Institute of Medical Sciences, Jodhpur, IND

**Keywords:** krebs von den lungen-6, c-reactive protein, severity, kl-6, neutrophilia, crp, chronic fibrosing interstitial lung diseases, bronchoalveolar lavage

## Abstract

Introduction

It is hypothesized that bronchoalveolar lavage (BAL) neutrophilia, Krebs von den Lungen-6 (KL-6), and C-reactive protein (CRP) predict the severity of chronic fibrosing interstitial lung diseases (CF-ILDs).

Methods

This cross-sectional study enrolled 30 CF-ILD patients. Using Pearson’s correlation analysis, BAL neutrophils, KL-6, and CRP were correlated with forced vital capacity (FVC), diffusing lung capacity for carbon monoxide (DLCO), six-minute walk distance (6MWD), partial pressure of oxygen (PaO2), computed tomography fibrosis score (CTFS), and pulmonary artery systolic pressure (PASP). Using the receiver operator characteristic (ROC) curve, BAL KL-6 and CRP were evaluated against FVC% and DLCO% in isolation and combination with BAL neutrophilia for predicting the severity of CF-ILDs.

Results

BAL neutrophilia significantly correlated only with FVC% (r = -0.38, P = 0.04) and DLCO% (r = -0.43, P = 0.03). BAL KL-6 showed a good correlation with FVC% (r = -0.44, P < 0.05) and DLCO% (r = -0.50, P = 0.02), while BAL CRP poorly correlated with all parameters (r = 0.0-0.2). Subset analysis of BAL CRP in patients with CTFS ≤ 15 showed a better association with FVC% (r = -0.28, P = 0.05) and DLCO% (r = -0.36, P = 0.04). BAL KL-6 cut-off ≥ 72.32 U/ml and BAL CRP ≥ 14.55 mg/L predicted severe disease with area under the curve (AUC) values of 0.77 and 0.71, respectively. The combination of BAL neutrophilia, KL-6, and CRP predicted severity with an AUC value of 0.89.

Conclusion

The combination of BAL neutrophilia, KL-6, and CRP facilitates the severity stratification of CF-ILDs complementing existing severity parameters.

## Introduction

Interstitial lung diseases (ILDs) encompass a large heterogeneous group of diseases associated with diverse underlying etiological factors and varying degrees of fibrosis and inflammation of lung parenchyma and/or interstitium [1]. The clinical course of chronic fibrosing interstitial lung diseases (CF-ILDs) is highly variable and unpredictable with few of them having a stable disease or gradual decline, whereas others progressing rapidly or being associated with frequent exacerbations [2]. Approximately 20-30% of CF-ILDs have a progressive course characterized by declining lung function, progressive fibrosis on chest high-resolution computed tomography (HRCT), deterioration of symptoms, reduced quality of life, suboptimal response to treatment, and increased mortality [3-5]. Therefore, predicting the severity and progression of CF-ILDs is paramount in their management. Although a plethora of information exists from pulmonary function tests, computed tomography (CT) thorax, and other physiological parameters to assess severity, their predictive capacity may be precluded by an insufficient respiratory effort, co-existent emphysema and pulmonary hypertension, or interobserver variability, thereby augmenting the need for novel investigation modalities in predicting severity, progression, and survival of CF-ILDs [6].

Bronchoalveolar lavage (BAL) cell differential counts have been underused in ILD severity. BAL neutrophilia has been correlated with disease severity and poor prognosis for chronic hypersensitivity pneumonitis (HP) and idiopathic pulmonary fibrosis (IPF), while BAL eosinophilia was associated with severe disease in patients with IPF [7-9].

Krebs von den Lungen-6 (KL-6) is a human high-molecular-weight glycoprotein encoded by the transmembrane glycoprotein mucin 1 (MUC1) gene and expressed on the outer surface of alveolar epithelial type II cells. It is believed to promote fibroblast migration and proliferation and is directly associated with the pathogenic process of ILD, reflecting the extent of damage and regeneration of type II pneumocytes [10]. High serum KL-6 levels have been found in IPF and other CF-ILDs. They correlate with increased severity and are a predictor of acute exacerbation [11,12]. Elevated BAL KL- 6 levels were found in patients with different ILDs, including IPF [13], but no study analyzed the significance of this elevation.

C-reactive protein (CRP), a homopentameric acute-phase reactant protein and part of the innate immune response to systemic inflammation, was found to be a predictive biomarker of disease severity and survival of ILD and identifying patients requiring intensive therapy [14]. Elevated baseline CRP levels correlated with severe disease in systemic sclerosis related-ILD (Ssc-ILD) [15]. The predictive implication of BAL CRP levels for ILD severity has not been studied so far.

Although serum biomarkers have recently come up in ILD diagnosis and management, there is a paucity of data on BAL biomarkers and their predictive ability to assess ILD severity. The present study, a first of its kind, aimed to find the clinical utility of BAL biomarkers in isolation and combination with BAL neutrophilia for predicting the severity of CF-ILDs in patients in whom aggressive management and early assessment for lung transplant can be done.

## Materials and methods

Study design and participants

This was a hospital-based, cross-sectional study carried out at All India Institute of Medical Sciences, Jodhpur, India, a tertiary-care teaching hospital, from June 2019 to June 2021. A total of 30 treatment-naïve adult CF-ILD patients were enrolled in the study after obtaining informed consent. Those presenting with acute exacerbation of ILD and co-existent chronic lung diseases, pulmonary infections, and malignancy were excluded. The study outcome was the diagnostic accuracy of BAL neutrophilia, KL-6, and CRP for predicting disease severity and their correlation with forced vital capacity (FVC), diffusing capacity of the lung for carbon monoxide (DLCO), six-minute walk distance (6MWD), partial pressure of oxygen (PaO2), pulmonary artery systolic pressure (PASP), and CT fibrosis score (CTFS). BAL KL-6 and high-sensitivity C-reactive protein (HS-CRP) were evaluated against lung function (FVC < 50% and DLCO < 35%) for predicting severe disease. To improve sensitivity, BAL CRP levels were measured by the HS-CRP test, as CF-ILDs usually include a predominant fibrotic phenotype with a mild degree of inflammation. The study was approved by the Institutional Ethics Committee, All India Institute of Medical Sciences, Jodhpur (AIIMS/IEC/2019/1810).

Data collection

Data were collected using a predesigned, semi-structured proforma. Data collection included baseline demographic, clinical, physiological, radiological, and laboratory data. Baseline investigations included routine blood investigations, pulmonary function tests, six-minute walk test (6MWT), arterial blood gases (ABG), transthoracic echocardiogram (TTE), chest radiograph, and HRCT thorax. BAL was performed as per the American Thoracic Society guidelines for ILD and analyzed for differential cell counts, KL-6, and CRP levels [16]. An integrated multidisciplinary discussion (MDD) involving a pulmonologist specialized in ILD, thoracic radiologists, and pathologists arrived at the final diagnosis of CF-ILDs after a careful review of data.

Definitions

Dyspnea severity was given by a modified medical research council scale [17]. Obesity was defined as a body mass index > 30 kg/m2 [18]. Respiratory failure was defined as PaO2 < 60 mmHg measured on room air. The severity classification of age, gender, FVC, and DLCO was given by the gender-age-physiology (GAP) index for prognostication of IPF [19]. Pulmonary hypertension was defined as PASP > 35 mmHg measured using the Bernoulli equation via TTE in an apical four-chamber view [20]. Smoke exposure was defined by a Brinkman Index ≥ 200 and/or significant exposure to biomass fuel/occupational/environmental smoke [21]. Semiquantitative CTFS in ILD proposed by Warrick et al. was used and classified into mild (score = 1-7), moderate (score = 8-15), and severe (score > 15) [22]. A 6MWD of less than 300 m was considered as having significant impairment in exercise capacity [23]. BAL neutrophilia/lymphocytosis/eosinophilia was defined as BAL neutrophils/lymphocytes/eosinophils > 5%, >15%, and >1% of BAL fluid cellular analysis, respectively [16]. If a mixed cellular pattern was observed on BAL, the predominant cellular pattern was considered.

Measurement of BAL KL-6 and CRP levels

BAL KL-6 and CRP were measured using the GENLISA ELISA Kit (Krishgen Biosystems, Mumbai, India) using the sandwich enzyme-linked immunosorbent assay (ELISA) technique. The kit employs dual monoclonal antibodies thereby improving the sensitivity and specificity. The limit of detection (LOD) of KL-6 ELISA was <18U/mL with intra-assay and inter-assay coefficient of variability (CV) of <15% and <18%, respectively. Whereas, the LOD of BAL HS-CRP was <0.112 ug/mL with intra-assay and inter-assay CV of <15% and <18%, respectively.

Sample size and statistical analysis

The sample size was estimated using the following formula [24]:

 $N=Z^{2}\frac{P(1-P)}{D^{2}}$

Where, Z = level of confidence, P = expected prevalence, and D = relative precision.

Prevalence data of CF-ILDs were extracted from the hospital's electronic records. The sample size was estimated to be 36 at a 95% confidence interval, 5% expected prevalence, and 10% relative precision. There was a decline in patient visits due to the COVID-19 pandemic and therefore, the sample size was limited to 30.

Numerical data were expressed as mean ± SD and categorical data were described using frequency and percentage. Pearson’s correlation analysis was used to study the correlation between BAL neutrophils, KL-6, and HS-CRP with FVC, DLCO, 6MWD, PaO2, PASP, and CTFS. The receiver operator characteristic (ROC) curve was plotted for BAL KL-6 and CRP for predicting FVC < 50% and DLCO < 35%. Microsoft Excel 2016 (Microsoft Corporation, Redmond, WA) and R version 4.11 (R Foundation for Statistical Computing, Vienna, Austria) were used for statistical analysis. All P-values were two-tailed and P < 0.05 was considered significant.

## Results

**Figure 1:**
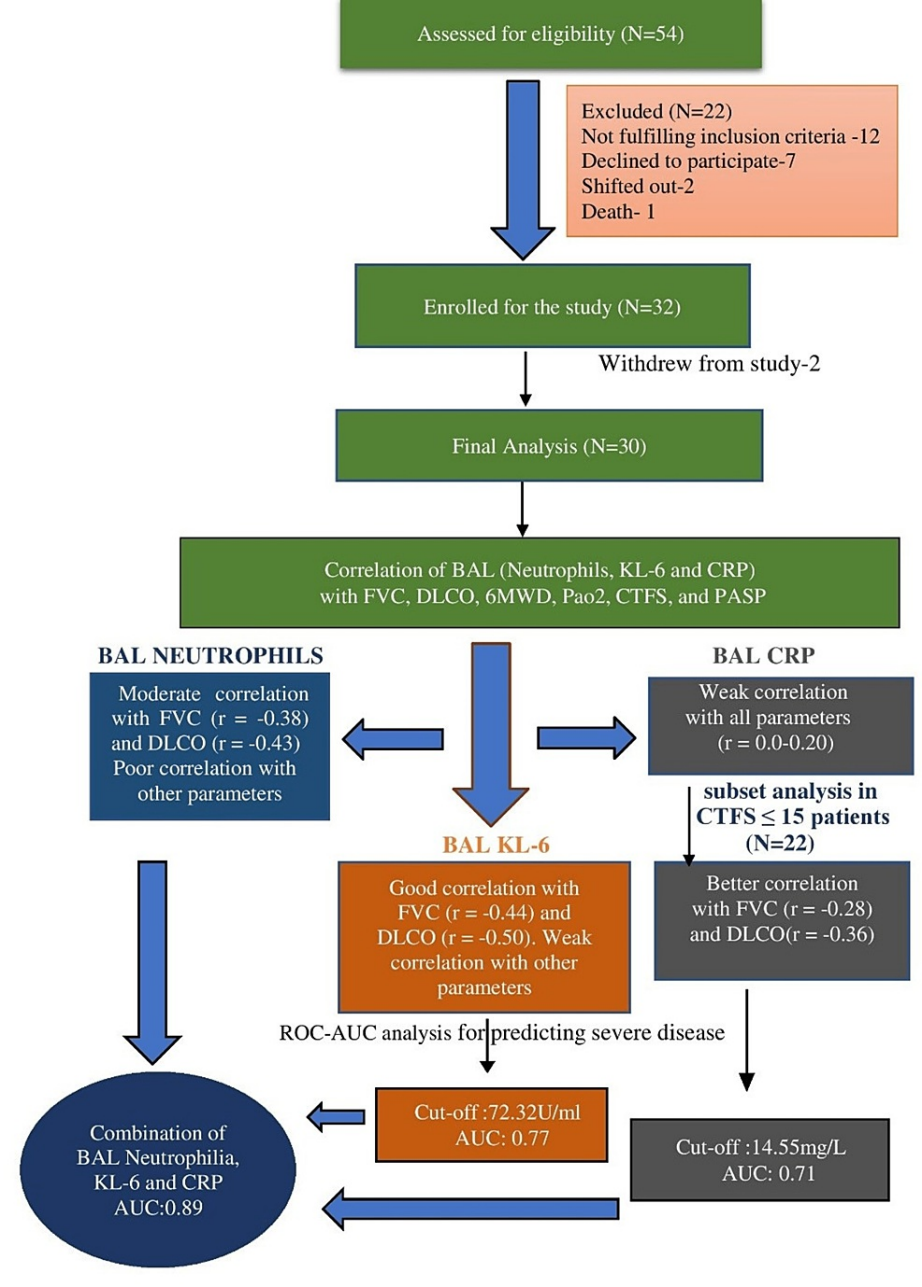
STROBE flow diagram of the study STROBE: Strengthening the Reporting of Observational Studies in Epidemiology; BAL: bronchoalveolar lavage; AUC: area under the curve; ROC: receiver operating characteristics; FVC: forced vital capacity; DLCO: diffusing lung capacity for carbon monoxide; 6MWD: six-minute walk distance; PaO2: partial pressure of oxygen; CTFS: computed tomography fibrosis score; PASP: pulmonary artery systolic pressure; KL-6: Krebs von den Lungen-6; CRP: C-reactive protein.

A total of 30 patients were enrolled. Figure 1 depicts the STROBE (Strengthening the Reporting of Observational Studies in Epidemiology) flow diagram of the study. The baseline characteristics of the study population are given in Table 1. The distribution of physiological and radiological severity parameters of the study participants is given in Table 2.

**Table 1:** Baseline characteristics of the study population BAL: bronchoalveolar lavage; GERD: gastroesophageal reflux disease; MDD: multidisciplinary discussion; HP: hypersensitivity pneumonitis; CTD-ILD: connective tissue disease-associated interstitial lung disease; IPF: idiopathic pulmonary fibrosis; NSIP: nonspecific interstitial pneumonia; ILD: interstitial lung disease.

Parameters	Number	Percentage
Age distribution in years (mean ± SD) = 50.33 ± 4.12		
18-40	06	20.00
41-60	19	63.33
>60	05	16.67
Gender		
Male	13	43.00
Female	17	57.00
Co-morbidities (n = 16)		
Systemic hypertension	05	20.00
Diabetes mellitus	04	13.33
Obesity	03	10.00
Ischemic heart disease	03	10.00
Hypothyroidism	01	03.33
Smoke exposure		
Present	10	33.33
Absent	20	66.67
Symptoms at presentation		
Dry cough	28	93.33
Dyspnea	30	100.0
Fatigue	06	20.00
Chest pain	03	10.00
Hemoptysis	01	03.33
Joint pain/swelling	04	13.33
Morning stiffness of joints	01	03.33
Raynaud phenomenon	02	06.67
GERD symptoms	08	26.67
Anorexia	05	16.67
Weight loss	03	10.00
Physical signs		
Pallor	04	13.33
Clubbing	09	30.00
Pedal edema	03	10.00
Elevated jugular venous pulse	05	16.67
Xerostomia/xerophthalmia	01	03.33
Oral ulcers	02	06.67
Cutaneous signs (hypopigmentation, skin rash, skin thickening, and digital ulcerations)	06	20.00
Joint tenderness/deformity	01	03.33
Velcro crepitations	23	76.67
Spirometry pattern		
Restrictive	26	86.67
Mixed	04	13.33
BAL cell differential counts		
Neutrophilia	12	40.00
Macrophage predominance	12	40.00
Lymphocytosis	06	20.00
Final diagnosis by MDD		
Fibrotic HP	08	26.67
Sarcoidosis	06	20.00
Interstitial pneumonia with autoimmune features	05	16.67
CTD-ILD	04	13.33
IPF	03	10.00
Idiopathic NSIP	02	06.67
Occupation-related ILD	02	06.67

**Table 2:** Distribution of physiological and radiological severity parameters in the study population

Parameters	No.	Percentage
Forced vital capacity %		
<50	23	76.67
≥50	07	23.33
Diffusing lung capacity for carbon monoxide %		
<35	09	30.00
≥35	21	70.00
Computed tomography fibrosis score		
1-7	04	13.33
8-15	18	60.00
>15	8	26.67
Six-minute walk distance (in meters)		
<300	14	46.67
≥300	16	53.33
Partial pressure of oxygen (in mmHg)		
<60	09	30.00
≥60	21	70.00
Pulmonary artery systolic pressure (in mmHg)		
>35	09	30.00
≤35	21	70.00

Pearson’s correlation analysis of BAL neutrophils showed a significant inverse correlation with FVC% (r = -0.38, P = 0.04) and DLCO% (r = -0.43, P = 0.03), and a weak correlation with CTFS (r = 0.12, P = 0.5), 6MWD (r = -0.04, P = 0.8), PASP (r = 0.12, P = 0.5), and PaO2 (r = -0.13, P = 0.5). BAL KL-6 showed a good correlation with FVC% (r = -0.44, P < 0.05) and DLCO% (r = -0.50, P = 0.02), and an insignificant correlation with PaO2 (r = -0.12, P = 0.52), PASP (r = -0.17, P = 0.36), CTFS (r = 0.18, P = 0.34), and 6MWD (r = 0.20, P = 0.28). There was a weak degree of correlation of BAL CRP with FVC% (r = -0.18, P = 0.05), DLCO% (r = -0.23, P = 0.04), PaO2 (r = -0.11, P = 0.56), 6MWD (r = -0.18, P = 0.34), PASP (r = -0.03, P = 0.87), and CTFS (r = 0.17, P = 0.36), while BAL CRP demonstrated better correlation with FVC% (r = -0.28, P = 0.05) and DLCO% (r = -0.36, P = 0.04) in the subset with mild to moderate CTFS (n = 22).

**Figure 2:**
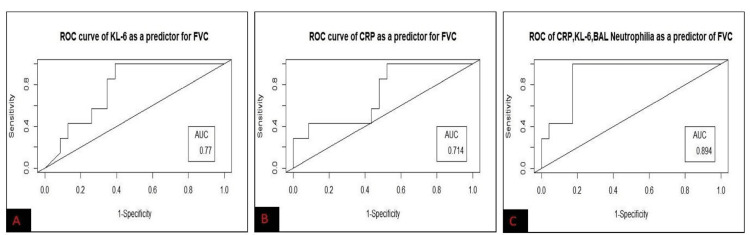
ROC curve of BAL KL-6 (A), BAL CRP (B), and a combination of BAL neutrophilia, KL-6, and CRP (C) for predicting low FVC in the study population BAL: bronchoalveolar lavage; AUC: area under the curve; ROC: receiver operating characteristics; FVC: forced vital capacity; KL-6: Krebs von den Lungen-6; CRP: C-reactive protein.

**Figure 3:**
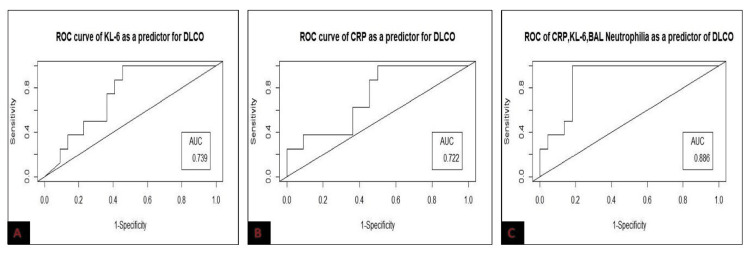
ROC curve of BAL KL-6 (A), BAL CRP (B), and a combination of BAL neutrophilia, KL-6, and CRP (C) for predicting low DLCO in the study population BAL: bronchoalveolar lavage; AUC: area under the curve; ROC: receiver operating characteristics; DLCO: diffusing capacity of the lungs for carbon monoxide; KL-6: Krebs von den Lungen-6; CRP: C-reactive protein.

The mean ± SD of BAL KL-6 of the study population was 73.64 ± 22.49 (range = 20-314.6) U/ml. In ROC curve analysis, BAL KL-6 cut-off value of 72.32 U/ml predicted FVC < 50% with a sensitivity of 68.4%, specificity of 100%, and area under the curve (AUC) value of 0.77. While BAL KL-6 cut-off ≥ 85.54 U/ml predicted DLCO < 35% with a sensitivity of 61.5%, specificity of 100%, and AUC value of 0.73. The mean ± SD of BAL CRP levels in the study group was 22.24 ± 8.37 mg/L. In ROC curve analysis, BAL CRP cut-off level of 14.55 mg/L was a significant predictor of FVC < 50% (sensitivity = 60%, specificity = 100%, and AUC = 0.72) and DLCO < 35% (sensitivity = 57.2%, specificity = 100%, and AUC = 0.71). The combination of BAL neutrophils, KL-6, and CRP predicted FVC < 50% and DLCO < 35% with an AUC value of 0.894 and 0.886, respectively (Figures 2, 3).

## Discussion

We recruited 30 treatment-naïve CF-ILD patients and analyzed the correlation of BAL neutrophils, KL-6, and CRP with existing parameters of disease severity. BAL cellular analysis revealed a neutrophilic BAL in 40% of the cases, lymphocytosis in 20%, and the remaining 40% had alveolar macrophage predominance. Neutrophils are innate immune cells and may contribute to lung fibrosis via fibroblast activation, extracellular matrix accumulation, as well as alveolar epithelial injury [25]. In a study by Salonen et al., BAL neutrophilia was found to be associated with shorter survival in ILD patients [26]. We assessed the correlation of BAL neutrophils with FVC, DLCO, 6MWD, PaO2, CTFS, and PASP and found a significant correlation only with FVC% (r = -0.38, P = 0.04) and DLCO% (r = -0.43, P = 0.03). This was consistent with a previous study by Kinder et al., who found BAL neutrophilia correlated with clinical-radiologic-physiologic severity score and early mortality [27]. In a study by Walters et al., BAL neutrophilia at diagnosis showed a significant inverse correlation with baseline DLCO in asbestosis-related ILD [28]. Haslam et al. [29] found that BAL neutrophilia correlated well with the degree of lung involvement, CTFS, and other parameters of disease severity in chronic HP patients. However, in our study, BAL neutrophils failed to show a significant correlation with CTFS (r = 0.12, P = 0.5). The discrepant finding in our study may be due to the fact that nearly two-thirds of the study participants had only mild to moderate degrees of parenchymal fibrosis (CTFS ≤ 15).

We examined whether BAL KL-6 could be used as a marker of disease severity in CF-ILDs. KL-6 is a high-molecular-weight glycoprotein expressed on type II pneumocytes and expresses alveolar damage caused by CD3+CD8+-mediated inflammation and has been demonstrated to play a key role in the pathogenesis of fibrotic lung diseases [29]. The mean distribution of BAL KL-6 in our study population was 73.64 ± 22.49 U/ml. This was relatively less compared to serum and BAL KL-6 levels in other previous studies [30,31]. This discordance may be attributed to the relatively lesser number of IPF patients (10%), as well as the exclusion of patients presenting with acute exacerbation in our study. This was reflected in the study by Zhu et al., who observed higher BAL and serum KL-6 levels in IPF compared with sarcoidosis, HP, and CTD-ILD [13]. KL-6 could serve as a specific severity marker of IPF and the heterogeneity of KL-6 values in IPF and non-IPF fibrotic ILDs is yet to be fully understood. Further, the majority of the studies on KL-6 were from East Asia (China, Korea, and Japan), and polymorphisms in the MUC1 encoding gene, as well as ethnicity, have been postulated to affect KL-6 levels [32]. Hence, validation of KL-6 levels in different subsets of the population is needed. ROC curve analysis of BAL KL-6 predicted severe disease (FVC < 50% and DLCO < 35%) and the optimum cut-off values were 72.32 and 85.54 U/ml, respectively. The cut-off value of 72.32 U/ml was used in our study, owing to its improved sensitivity and better diagnostic accuracy with regard to predicting severe disease.

There was a significant inverse correlation of BAL KL-6 levels with lung function (FVC% (r = -0.44, P < 0.05) and DLCO% (r = -0.50, P = 0.02)) while the degree of correlation was poor with the other parameters. A prospective study from South Korea showed a significant correlation of KL-6 with FVC, DLCO, and CTFS [33]. Similar results were seen in the study by Qin et al., where serum KL-6 levels significantly correlated with DLCO% (r = -0.51, P < 0.00) and CT scores (r = 0.53, P = 0.00) [34]. A retrospective study by Kim et al. showed that KL-6 levels were inversely correlated with lung function and exercise capacity [35]. An exploratory study by Bennett et al. [36] found BAL KL-6 was inversely correlated with baseline lung function and 6MWD. Therefore, KL-6 may be considered as a predictive marker of disease severity in CF-ILDs.

We aimed to find if BAL CRP could predict severity in CF-ILDs. There is a lacuna of data regarding its utility as a biomarker in ILD and to the best of our knowledge, our study is the first to assess its role in CF-ILDs. We chose to evaluate BAL CRP in CF-ILDs, as there may be heterogeneity in the degree of inflammation and fibrosis in these subsets of ILD and could benefit from potential initiation/cessation of anti-inflammatory and/or antifibrotic therapy. ROC curve analysis of BAL CRP for predicting disease severity (FVC < 50% and DLCO < 35%) yielded an optimum cut-off value of 14.55 mg/L, with an AUC value of 0.72 and 0.71, respectively.

BAL CRP showed a significant correlation only with FVC% (r = -0.18, P = 0.05) and DLCO% (r = -0.23, P = 0.04), albeit a low degree of correlation. Our results are in agreement with a prospective study by Liu et al., who found elevated CRP levels correlated with low FVC and DLCO [24]. Also, the results from a multicenter study showed a negative correlation of CRP with lung function and a positive correlation with PASP and CTFS [15]. A study by Robles-Pérez et al. showed elevated CRP levels inversely correlated with DLCO and FVC [36]. The lower degree of correlation of BAL CRP in our study may be ascribed to the varying severity of CTFS in our patients. Therefore, we performed correlation analysis in the subset of patients with mild to moderate CTFS (≤15) and found considerable improvement in the degree of correlation of BAL CRP with lung function (FVC% (r = -0.28, P = 0.05) and DLCO% (r = -0.36, P = 0.04)) as well as a significant negative correlation with CTFS (r = -0.22, P = 0.05). BAL CRP may play a significant role in predicting the degree of inflammation in CF-ILD patients with mixed inflammatory and fibrotic phenotypes who may benefit from glucocorticoid and immunosuppressive therapy.

We found that the combination of BAL neutrophilia and biomarkers lead to an improvement in diagnostic accuracy of predicting severe diseases rather than viewing them in isolation. This is consistent with the findings of other previous studies [37,38]. BAL neutrophilia and the two biomarkers can be useful for facilitating severity stratification of CF-ILDs, thereby identifying patients requiring urgent aggressive therapy and referral for a lung transplant.

This study was carried out in a single tertiary-care referral center. Hence, these results cannot be generalized, and also owing to the relatively small sample size, the study participants may not be representative of all phenotypes of CF-ILDs.

## Conclusions

The combination of BAL neutrophilia and biomarkers might serve as a useful adjunct to predict the severity of CF-ILDs. Baseline BAL cell differential counts and biomarker level measurements, wherever feasible, may be included in the initial assessment of CF-ILDs. The BIO-FILD study adds impetus to the evolving spectrum of evidence for BAL biomarkers in the management amalgam of CF-ILDs. Hitherto, future large-scale multicentric studies with diverse population subsets are required to validate our findings before incorporating them into routine clinical practice.
